# Evolution of chemokine receptors is driven by mutations in the sodium binding site

**DOI:** 10.1371/journal.pcbi.1006209

**Published:** 2018-06-18

**Authors:** Bruck Taddese, Madeline Deniaud, Antoine Garnier, Asma Tiss, Hajer Guissouma, Hervé Abdi, Daniel Henrion, Marie Chabbert

**Affiliations:** 1 Laboratoire MITOVASC, UMR CNRS 6015 – INSERM 1083, Université d’Angers, Angers, France; 2 Laboratoire de Génétique, Immunologie et Pathologies Humaines, Faculté des Sciences de Tunis, Université de Tunis El Manar, Tunis, Tunisie; 3 The University of Texas at Dallas, School of Behavioral and Brain Sciences, Dallas, Texas, United States of America; Max Planck Institute for Biophysical Chemistry, GERMANY

## Abstract

Chemokines and their receptors (members of the GPCR super-family) are involved in a wide variety of physiological processes and diseases; thus, understanding the specificity of the chemokine receptor family could help develop new receptor specific drugs. Here, we explore the evolutionary mechanisms that led to the emergence of the chemokine receptors. Based on GPCR hierarchical classification, we analyzed nested GPCR sets with an eigen decomposition approach of the sequence covariation matrix and determined three key residues whose mutation was crucial for the emergence of the chemokine receptors and their subsequent divergence into homeostatic and inflammatory receptors. These residues are part of the allosteric sodium binding site. Their structural and functional roles were investigated by molecular dynamics simulations of CXCR4 and CCR5 as prototypes of homeostatic and inflammatory chemokine receptors, respectively. This study indicates that the three mutations crucial for the evolution of the chemokine receptors dramatically altered the sodium binding mode. In CXCR4, the sodium ion is tightly bound by four protein atoms and one water molecule. In CCR5, the sodium ion is mobile within the binding pocket and moves between different sites involving from one to three protein atoms and two to five water molecules. Analysis of chemokine receptor evolution reveals that a highly constrained sodium binding site characterized most ancient receptors, and that the constraints were subsequently loosened during the divergence of this receptor family. We discuss the implications of these findings for the evolution of the chemokine receptor functions and mechanisms of action.

## Author summary

The chemokine-receptor system is involved in a broad array of pathologies, including autoimmune and inflammatory diseases, allergies, cancer metastasis, and HIV infection. It is an attractive, but difficult, target for drug development and a deeper understanding of the structure-function relationships of the chemokine receptors is required to help design drugs targeted against these receptors. To gain information on the mechanism of action of the chemokine receptors, we developed an evolutionary approach based on the global analysis of co-mutations in receptor sequences. This approach drew attention to a few residues whose mutation was crucial for the evolution of the chemokine receptor family. To understand the role of these residues, we have carried out molecular dynamics simulations that revealed that these mutations dramatically modified the binding mode of a sodium ion involved in receptor regulation. These changes accompanied the divergence of chemokine receptor functions between immune surveillance and inflammation. They indicate unanticipated roles of the sodium ion in the mechanism of action of the chemokine receptors.

## Introduction

Directed cell migration is fundamental for life because this process is involved in key biological processes such as embryonic development, organogenesis, immune surveillance, host defense, and wound repair. Leukocyte migration and tissue localization during homeostatic and inflammatory conditions depend directly upon chemokines (or chemotactic cytokines), a family of small secreted proteins. Chemokines implement their functions by acting through specific receptors belonging to the family of class A (rhodopsin-like) G-protein-coupled receptors (GPCRs). The human chemokine-receptor system is composed of forty-five chemokines and twenty-two receptors, with complex specificity/promiscuity pattern [1]. Some chemokine-receptor pairs are highly specific but most chemokines and receptors can be involved in different pairings. This system has a highly positive developmental and protective role in physiological conditions but it is also implicated in a broad array of pathologies, including autoimmune and inflammatory diseases, allergies, cancer metastasis, and HIV infection. Therefore, the chemokine-receptor system is an attractive target for drug development [1, 2]. Among the chemokine receptors, CXCR4 and CCR5 have been extensively studied because of their role as co-receptors of HIV for virus entry.

Chemokines are structurally classified as CC, CXC, C3XC, and C chemokines, based on the arrangement of the N-terminal disulfide forming cysteines. Chemokines can also be classified according to their main function [3]. The homeostatic chemokines are involved in homing of lymphocytes in physiological conditions, whereas inflammatory chemokines are involved in attracting lymphocytes in inflammatory area (note that some chemokines have dual functions). Chemokine receptors can be classified by phylogeny into two groups. The oldest group, which appeared in jawless fishes, binds mainly homeostatic chemokines while the most recent group, which appeared in jawed vertebrates, bind mainly inflammatory chemokines [3, 4]. The “atypical” chemokine receptors with promiscuous chemokine binding are phylogenetically related to either one of these groups. Previously called decoys or scavengers, these atypical receptors usually act as β-arrestin biased receptors that do not promote migration but rather shape chemokine gradients to permit migration induced by conventional chemokine receptors [5].

Several structures of chemokine receptors, in inactive or pseudo-active forms, bound to chemical ligands or chemokines, have been resolved [6–11] and have provided invaluable information on the mechanism of action of these receptors. Details of the mechanism of chemokine binding to cognate receptors are emerging with the analysis of the recent structures of chemokine-receptor complexes [6, 9]. The structure of these complexes corroborates the insertion of the chemokine N-terminus into the receptor helical core and the plasticity of the chemokine receptors to adapt to different ligands.

Because of the wide variety of diseases in which chemokine receptors are implicated, chemokine receptors constitute very attractive targets for the pharmaceutical industry. However, despite important investments, only two drugs targeting chemokine receptors have received Food and Drug Administration approval for clinical use: maraviroc, which targets CCR5 in HIV/AIDS treatment, and plerifaxor, which targets CXCR4 for hematopoietic stem cell mobilization. Difficulties in targeting chemokine receptors for anti-inflammatory therapy may arise from inappropriate target selection and ineffective dosing or from the redundancy of the chemokine system [12]. Recent advances in the understanding of chemokine signaling have shown that this apparent redundancy hides biased signaling. The activation of the same receptor by different chemokines may induce different cellular issues [13]. This observation indicates a system more complex than initially thought. In addition, the effect of a ligand may depend on the presence of different chemokines and of the cellular system or tissue under investigation [1]. Finally, the chemokine/receptor system is species-specific and may lead to different results in mouse/rat trials compared to humans. Taken together, these additional levels of complexity make pharmacodynamics and pharmacology studies very difficult for therapeutic applications.

Understanding the molecular determinants involved in functional specificity of chemokine receptors could help the rational design of drugs targeted towards this important receptor sub-family. Evolutionary information, based on analysis of multiple sequence alignment (MSA) can be used to gain structural and functional information on protein families. Previously, we have used evolutionary information to successfully predict the kinked structure of the transmembrane helix 2 (TM2) in chemokine receptors prior to their crystallization [14]. These receptors are part of a larger sub-family, the chemotaxic (CHEM) sub-family in Fredriksson’s classification, which includes different chemotaxic and vasoactive receptors [15]. We have shown that the CHEM sub-family, along with the PUR sub-family of purinergic receptors, evolved by divergence from the somatostatin/opioid (SO) receptor sub-family in vertebrates, and that the latter sub-family evolved from the deletion of one residue in TM2 in an ancestral receptor [14, 16]. Receptors from these three sub-families possess a characteristic P2.58 pattern (Ballesteros’ numbering) which corresponds to one of the main GPCR evolutionary pathways [16, 17].

In the present study, we investigate the evolutionary determinants of chemokine receptors using principal component analysis of sequence covariations in nested GPCR sequence sets. This approach highlights three residues whose mutations were crucial for the emergence of chemokine receptors and their subsequent divergence into homeostatic and inflammatory receptors. These key residues are located at the binding site of a sodium ion which is thought to be a general feature amongst class A GPCRs [18, 19]. To further define the structural/functional role of these residues, we carried out molecular dynamics (MD) simulations of the chemokine receptors CCR5 and CXCR4, chosen as prototypes of homeostatic and inflammatory chemokine receptors. We show that the evolution of chemokine receptors was driven, at least in part, by dramatic changes in the sodium binding mode. Most ancient receptors, which appeared in jawless fishes, have a highly constrained sodium binding site. These constraints were subsequently loosened during the divergence of this receptor family. We discuss the implications of these findings in terms of evolution of the chemokine receptor functions and mechanisms of action.

## Results

### Principal component analysis reveals key residues of the chemokine receptor sub-family divergence

**Fig 1:**
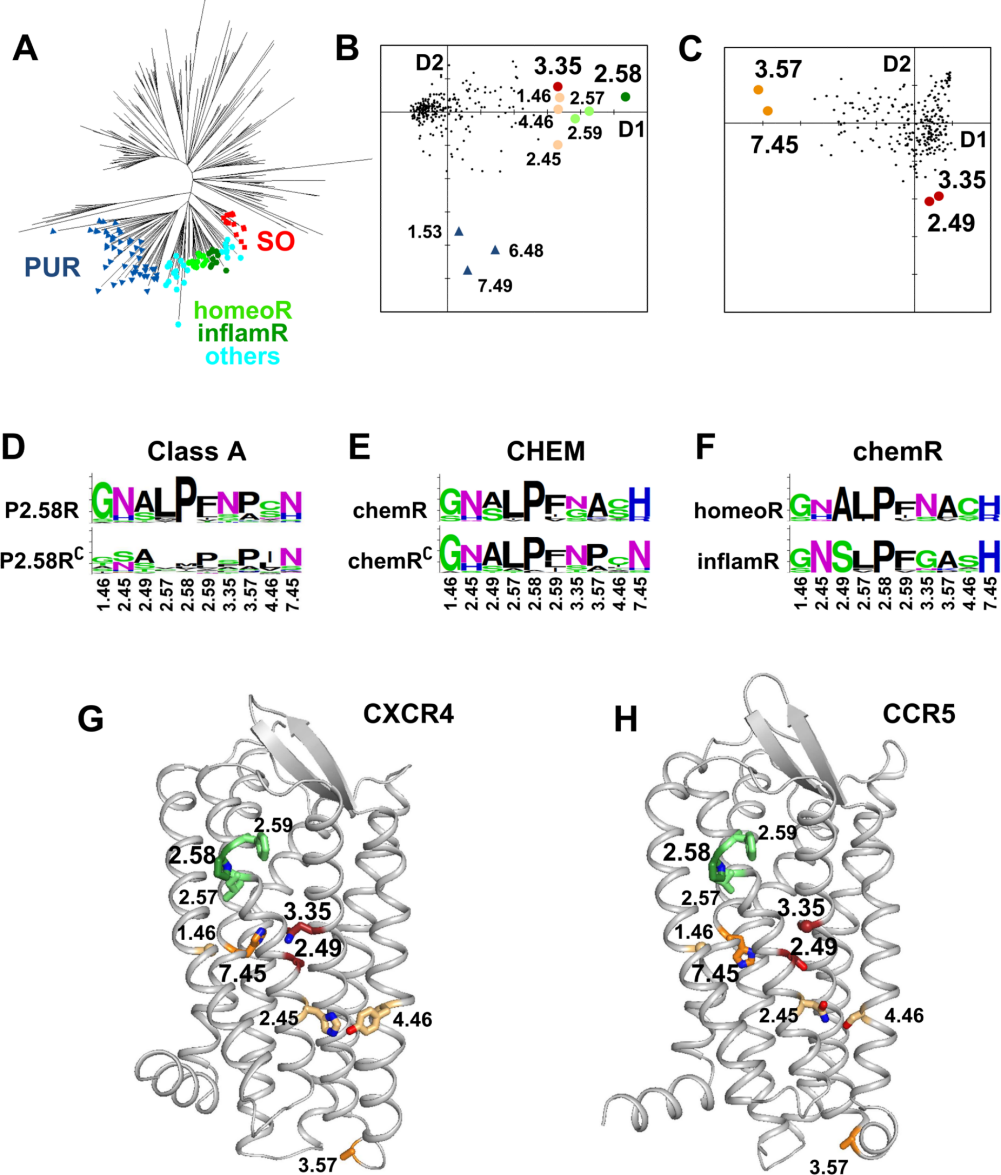
Positions characterizing chemokine receptors. (A) NJ tree of human class A GPCRs. The three sub-families forming the P2.58 set are indicated by labels. SO and PUR receptors are indicated, respectively, by red squares and blue triangles. The CHEM receptors are indicated by circles. The homeostatic and inflammatory chemokine receptors are colored, respectively, with bright green and dark green. The other CHEM receptors are colored in cyan. (B, C) PCA analysis of the covariation matrix obtained from the MSA of the human class A GPCRs (B) and of the human CHEM sub-family (C). The positions in the alignment are projected onto the first two dimensions of the PCA. The coordinates on the first and second axes are indicative of the covariation scores. The positions characterizing the main evolutionary pathways (largest coordinates on the first and second axes) are indicated by large circles. In (B), large circles indicate the positions that most covary with position 2.58. Position 2.58 is dark green, positions 2.57 and 2.59 are light green, position 3.35 is dark red and positions 1.46, 2.45 and 4.46 are salmon. Blue triangles indicate the positions (7.49, 6.48, and 1.53) that most covary with the divergence between CHEM and PUR receptors. In (C), orange circles indicate the positions that most covary with the divergence of chemokine receptors (3.57 and 7.45) and dark red circles indicate the positions that most covary with the divergence between homeostatic and inflammatory receptors (2.49 and 3.35). (D-F) Logo plots of positions that are highlighted by PCA analysis in different GPCR sets. In (D), human class A GPCRs are split into P2.58 receptors (top) and the complementary sequences (bottom). In (E), the human CHEM sub-family is split into chemokine receptors (top) and the complementary sequences (bottom). In (F), the human chemokine receptors (chemR) are split into homeostatic (top) and inflammatory chemokine receptors (bottom). (G, H) Ribbon representation of CXCR4 (G) and CCR5 (H), with highlighted positions shown as sticks. The color code is the same as in plots (B) and (C). The CXCR4 and CCR5 models are based on the PDB entries 3ODU and 4MBS, respectively, as described in Methods.

To highlight residues characterizing chemokine receptors, we applied a hierarchical approach to search residues characteristic of the four nested sets of human sequences that lead from class A receptors to the chemokine receptor sub-family. These sets correspond to: (1) class A GPCRs, (2) the P2.58 receptors (SO, CHEM and PUR sub-families), (3) the CHEM sub-family and (4) the chemokine receptor sub-family. Sequence sets were prepared as described in Methods. They are visualized on the Neighbor Joining (NJ) tree of human receptors shown in Fig 1A.

In a previous study [17], we have analyzed the sequence covariation in the multiple sequence alignment (MSA) of human class A GPCRs. The network representation of the top pairs with highest covariation scores highlighted the central role of position 2.58 as an evolutionary hub. This representation provides information on positions that covary with the P2.58 pattern. However, this arrangement is dependent on the number of top pairs selected. To obtain a representation of the covariation data independent of a user selected parameter, we carried out the principal component analysis (PCA) [20] of the double-centered covariation matrix (specifically, an eigen-decomposition of this matrix), obtained from the MSA of human class A GPCRs (S1 File). Fig 1B shows the positions in the MSA plotted in the plane formed by the first two components of the PCA. This analysis highlights a few positions clearly separated on the first and the second axes. The position with the highest coordinate on the first component is 2.58. Next are positions 2.57 and 2.59, then positions 1.46, 3.35, 4.46, and 2.45. These positions have the top covariation scores with position 2.58 [17]. On the second dimension, residues with highest coordinates are positions 7.49, then 6.48 and 1.53. These positions correspond to hallmark residues that led to the divergence of the PUR sub-family. Indeed, positions 7.49 and 6.48 are highly conserved Asn and Trp in most class A GPCRs, but are Asp and Phe in the PUR sub-family. Likewise, position 1.53 is usually Val in most GPCRs but Ala in PUR receptors.

Proline 2.58 is the hallmark residue of the SO, CHEM, and PUR sub-families. This pattern results from the deletion of one residue located two positions upstream of the TM2 proline in an ancestral P2.59 receptor [14]. The covariation of positions 2.57 and 2.59 with position 2.58 is a consequence of the indel mechanism. Logo representation of amino acid distribution (Fig 1D) indicates an increase in the frequency of Gly and Asn at positions 1.46 and 2.45, respectively, and a small polar residue instead of an aliphatic residue at position 4.46 in P2.58 receptors. In addition, position 3.35 is Asn in most P2.58 receptors while this amino acid is absent at this position in the complementary set.

The next step was to apply the same approach to the MSA of the human CHEM sub-family (S2 File). This analysis (Fig 1C) highlights residues associated with the divergence of chemokine receptors on the first component and the split between homeostatic and inflammatory receptors on the second component. Position 3.57, at the limit between TM3 and ICL2, is an alanine in chemokine receptors and a proline in other CHEM receptors (Fig 1E). However, we can note that the presence of Pro or Ala at this position is a common feature of human class A GPCRs (Fig 1D), a pattern which suggests a role in the interaction with G proteins. Most interestingly, position 7.45 in TM7 is either His or Arg in chemokine receptors, which is very infrequent in other human GPCRs (2%). Position 7.45 is preferentially Asn in class A receptors (67%). The second component highlights positions 2.49 and 3.35. We have previously shown that position 2.49 differentiates homeostatic and inflammatory receptors (A2.49 and S2.49, respectively) [17]. This position is strongly correlated with position 3.35, which is preferentially Asn and Gly in homeostatic and inflammatory receptors, respectively (Fig 1F).

The position of these hallmark residues in the structure of CXCR4 and CCR5, as prototypes of homeostatic and inflammatory chemokine receptors, is displayed in Fig 1G and 1H. Polar positions 2.45 and 4.46 are located on the external surface of the receptor at the interface with the membrane. These two positions face each other and may be involved in polar interactions. Indeed, in the crystal structure of CCR5, S4.46 is involved in H-bonding with N2.45. Most interestingly, positions 2.49, 3.35, and 7.45 are clustered in the receptor core and line the allosteric sodium binding pocket. Among them, positions 3.35 and 7.45 can be directly involved in the coordination of the sodium ion [18].

### History of the sodium binding site

In the two crystal structures of sodium bound P2.58 receptors, the δ-opioid receptor (OPRD, PDB entry 4N6H) [21] and the proteinase activated receptor 1 (PAR1, PDB entry 3VW7) [22], positions 3.35 and 7.45 participate in sodium binding, either directly (N3.35 in OPRD) or through a water molecule (N7.45 in OPRD and N3.35/S7.45 in PAR1). This ion acts as a negative modulator of GPCRs and stabilizes the inactive structure [18]. Mutations of N3.35 to smaller residues (Ser or Ala) in CXCR4 [23] and CXCR3 [24] yield constitutively active mutants. In the closely related angiotensin II receptor AT1, the N3.35G mutant has also high constitutive activity [25]. The presence of a glycine residue at position 3.35 in inflammatory chemokine receptors is thus surprising, and this prompted us to investigate the history of position 3.35.

Covariation can result either from phylogenetic history with the correlated residues already present in the common ancestor (or in an early step of subsequent evolution) and maintained throughout evolution or from an epistasis mechanism in which several correlated mutations led to functional divergence in the receptor family. Differentiation between the two mechanisms requires the analysis of the GPCR repertoires from different species covering several animal phyla.

**Fig 2:**
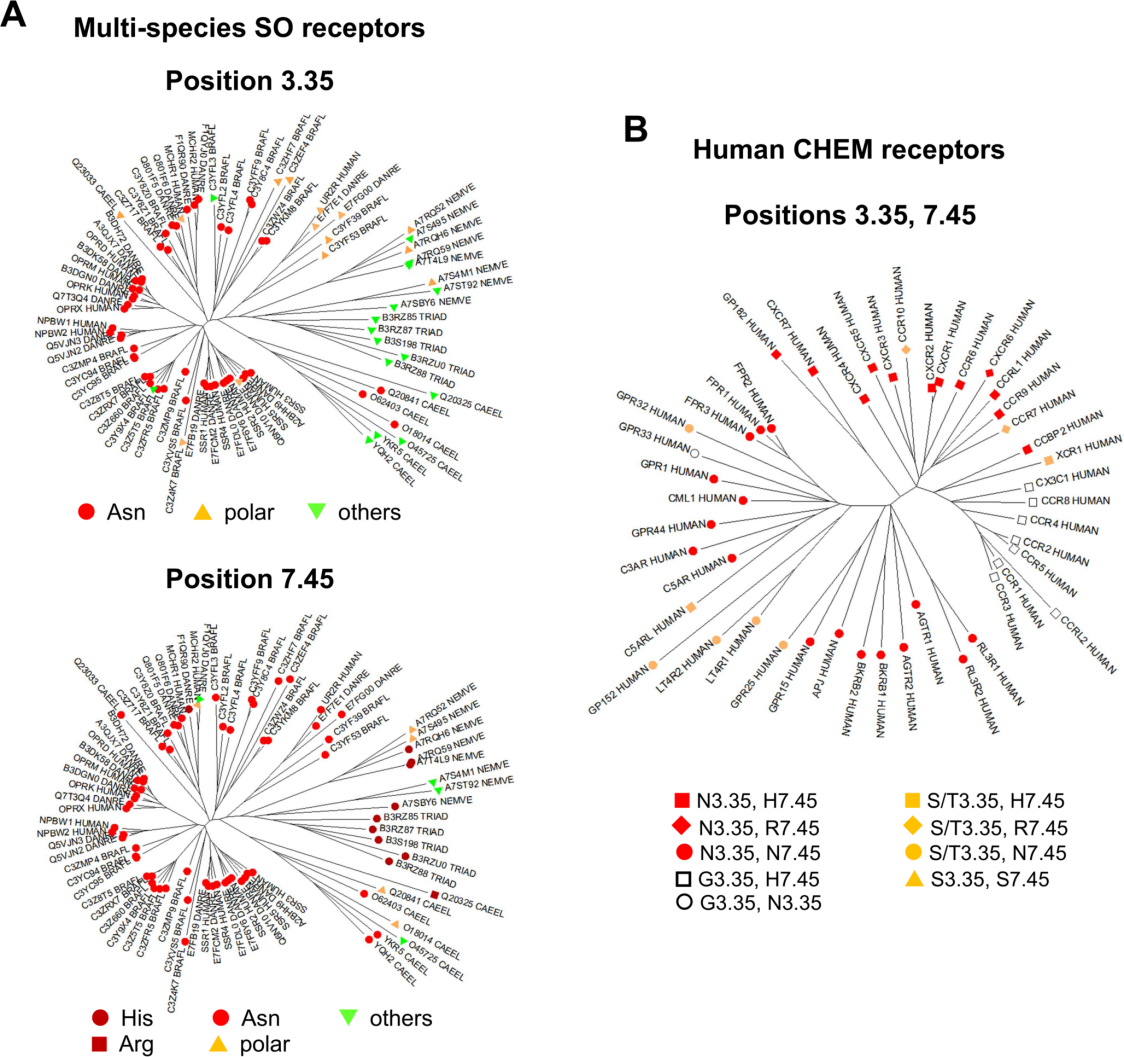
Evolution of the sodium binding site. (A) NJ tree of SO receptors from *H*. *Sapiens* (HUMAN), *D rerio* (DANRE), *B*. *floridae* (BRAFL), *C*. *elegans* (CAEEL), N. *vectensis* (NEMVE), and *T*. *adhaerens* (TRIAD). In the top panel, the labels indicate the sequence pattern at position 3.35 (Asn: red circles, other polar residues: orange triangles, apolar residues: green triangles). In the bottom panel, the labels indicate the sequence pattern at position 7.45 (His: dark red circles, Arg: dark red squares, Asn: red circles, other polar residues: orange triangles, apolar residues: green triangles); (B) NJ of the CHEM receptors from *H*. *sapiens*. The colors indicate the residue at position 3.35 (Asn: red, Ser/Thr: orange, Gly: white). The labels indicate the residue at position 7.45 (His: squares, Arg: diamonds, Asn: circles, Ser: triangles). The sequences are named by their Uniprot identifiers.

The CHEM and PUR sub-families are specific to vertebrate species [14, 16]. The N3.35 pattern is present in vertebrate SO receptors as exemplified by the opioid receptors. Thus, we analyzed the amino acid distribution at position 3.35 in the SO sub-family from different species: *H*. *sapiens*, *D*. *rerio*, *B*. *floridae*, *C*. *elegans*, *N*. *vectensis* and *T*. *adhaerens* (S3 File) and reported it on the NJ tree of these receptors (Fig 2A). The N3.35 pattern is not present in the receptors from non bilaterian species (*N*. *vectensis* and *T*. *adhaerens*), but polar residues (S, T) at this position are observed in sequences from *N*. *vectensis*. The N3.35 pattern is present in a few sequences from *C*. *elegans*, and in almost all chordates sequences. Interestingly, orthologs of the urotensin II receptor (UR2R in Uniprot nomenclature) are present in *B*. *floridae*. This is the first observation of UR2R in an invertebrate species. The small sub-family containing UR2R (Fig 2A) is characterized by the T3.35 pattern and is more closely related to invertebrate SO receptors, as an intermediate between chordate and non-chordate SO members. This analysis indicates that the N3.35 pattern, which strongly covaries with the P2.58 pattern in human GPCRs, is a hallmark of chordate SO receptors. The presence of N3.35 is correlated with the evolutionary drift of SO receptors observed by multidimensional scaling [16], a pattern suggesting that this residue might have contributed to the evolution of the SO sub-family and its subsequent divergence. This residue, directly involved in the binding of the allosteric sodium ion in the δ-opioid receptor [21], is present in most vertebrate SO, CHEM, and PUR receptors. This study strongly supports the assumption that the N3.35 pattern is secondary to the deletion in TM2 and might have been important for the evolutionary drift of P2.58 receptors in vertebrates.

We also analyzed position 7.45 in the same set of SO receptors (Fig 2A). In most receptors, position 7.45 is polar. This polar residue is usually Asn in chordates SO receptors (*B*. *floridae*, *D*. *rerio*, *H*. *sapiens*), with a single observation of His in a sequence from *D*. *rerio*. This is not the case in sequences from non-chordate species in which position 7.45 is more variable with several examples of His in *T*. *adhaerens* and *N*. *vectensis* and an example of Arg *in C*. *elegans*.

Finally, we analyzed positions 3.35 and 7.45 in the human CHEM sub-family (Fig 2B). The H7.45 pattern is a hallmark of chemokine receptors, indicating that the mutation of this position may have been crucial for the emergence of chemokine receptors. H7.45 is found in 20 out of 23 chemokine receptors, while R7.45 is found in only three homeostatic receptors (CXCR6, CCR7, and CCR10). Among other CHEM receptors, position 7.45 is usually Asn (84%) and the H7.45 pattern is observed only in the orphan GPR182, closely related to ACKR3/CXCR7 and in C5aRL. In chemokine receptors, position 3.35 is Asn in 10 out of 12 homeostatic receptors and Gly in 8 out of 10 inflammatory receptors. In the other CHEM receptors, position 3.35 is either Asn (72%) or Ser/Thr (24%) and Gly is observed only in the orphan GPR33 receptor.

### Dramatic changes in the sodium binding mode of CXCR4 and CCR5

**Fig 3:**
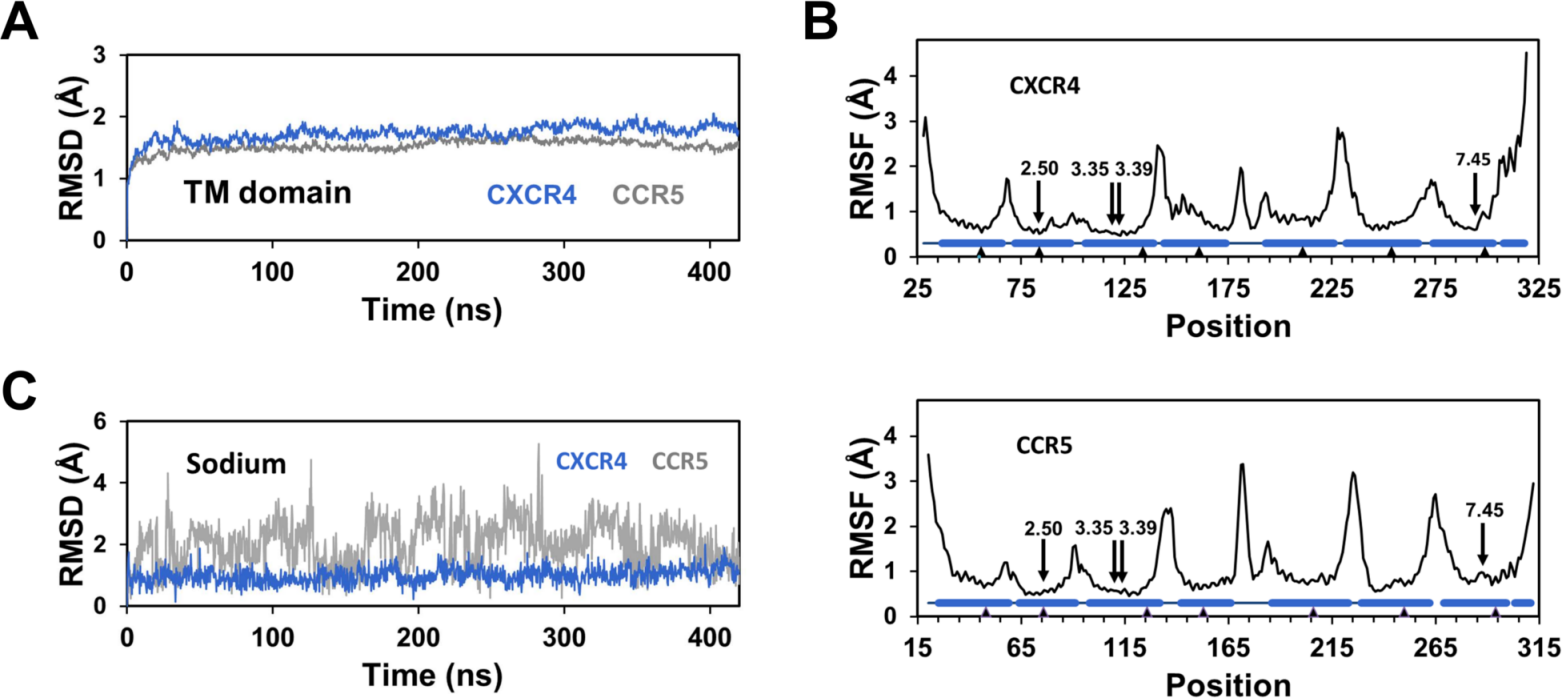
Stability of the CXCR4 and CCR5 receptors and of the bound sodium ion during MD simulations. (A) Root mean squared deviations (RMSD) of the Cα atoms of the TM helices are shown in blue and grey for CXCR4 and CCR5, respectively; (B) Root mean square fluctuations (RMSF) of the Cα atoms are shown in the top and bottom panels for CXCR4 and CCR5, respectively. The positions of the seven TM helices and of the C-terminal helix 8 are indicated as barrels. The anchor positions in Ballesteros’ numbering are represented by triangles. The arrows highlight the positions of the residues lining the sodium binding pocket. The data in (A) and (B) represent the average of the replicates. (C) Typical RMSD of the sodium ion in representative MD simulations are shown in blue and grey for CXCR4 and CCR5, respectively.

To analyze the consequences of these mutations on the mechanism of sodium binding, we carried out molecular dynamics simulations of CXCR4 and CCR5 in the presence of a sodium ion which was initially positioned in the receptor models in the vicinity of D2.50 (see Methods). After insertion of the models within a hydrated POPC bilayer, MD simulations were carried out for 420 ns. In both cases, the root mean square deviations (RMSD) of the Cα atoms of the transmembrane (TM) domain underwent a very fast increase of about 1 Å within the first nanosecond, followed by a slower phase that reached a plateau at about 1.7 Å after 20 to 40 ns (Fig 3A). The root mean square fluctuations (RMSF) indicated similar magnitude of fluctuations for both receptors. As usual in GPCRs, the RMSF of the loops and the termini could reach 3–4 Å, whereas the residues in the central part of the TM helices had fluctuations below 1 Å (Fig 3B). We can note that (1) the presence of three glycine residues in TM3 of CCR5, at positions 3.30, 3.35 and 3.39, does not alter the fluctuations of this helix as compared to CXCR4, and (2) the positions lining the sodium binding pocket (residues 2.50, 3.35, 3.39, and 7.45) have similar low fluctuations in both receptors. However, in spite of these similarities, striking differences were observed in the behavior of the sodium ion bound to CXCR4 and CCR5 during the simulations (Fig 3C). In CXCR4, after fast motion during the first nanosecond, the sodium ion remained stable with an average RMSD of 1.3 ± 0.3 Å. Similar results were seen for the three CXCR4 replicates. By contrast, in CCR5, the RMSD of the sodium ion did not converge but indicated exchanges between (at least) two positions with RMSD of approximately 1.2 and 2.5 Å. These exchanges provided different RMSD patterns for the five CCR5 replicates carried out.

**Fig 4:**
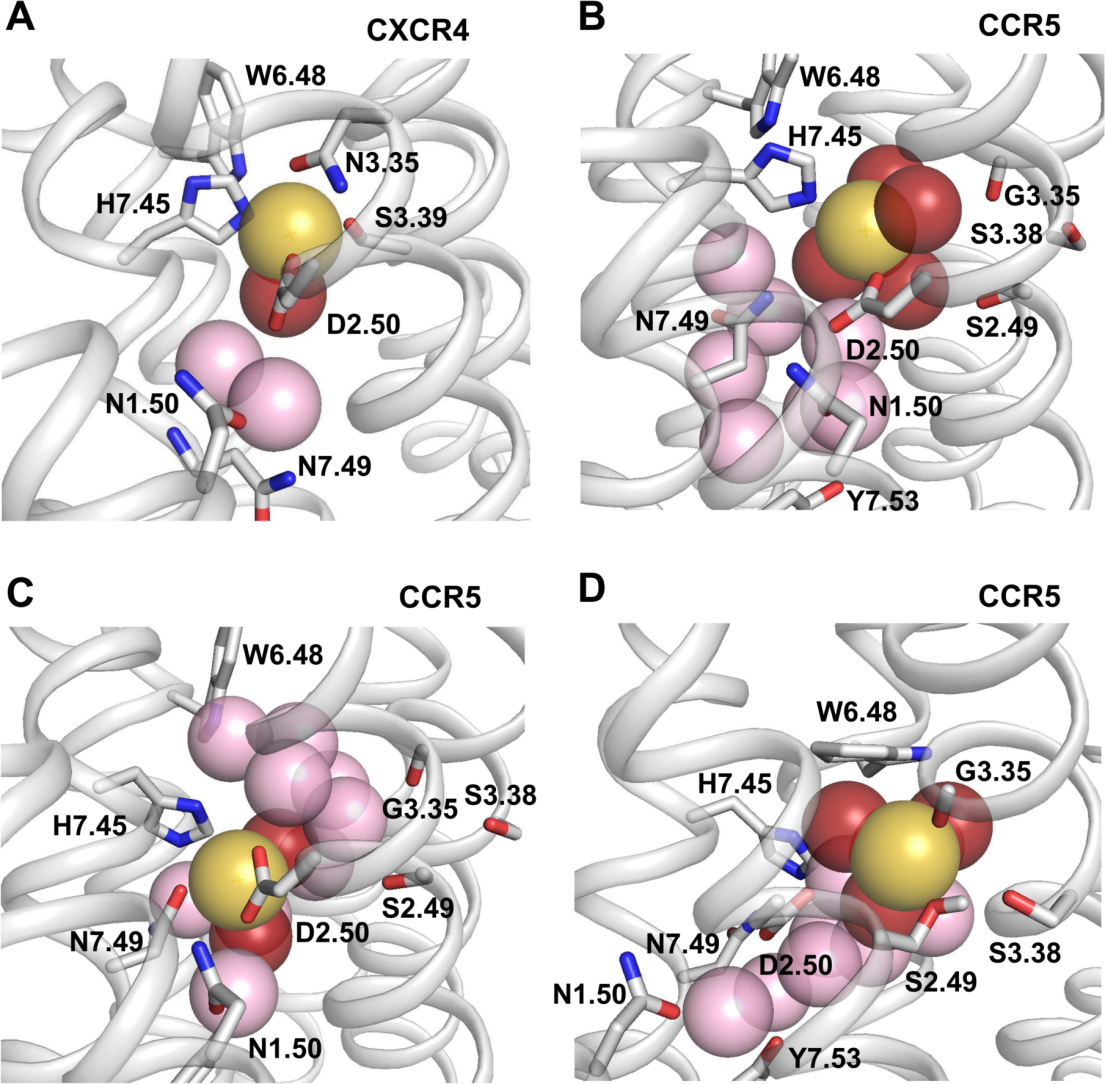
Representative snapshots of the sodium binding site in CXCR4 and CCR5. In (A), the sodium ion bound to CXCR4 interacts with D2.50, N3.35, S3.39, H7.45, and a water molecule. In (B), the sodium ion bound to CCR5 interacts with D2.50, H7.45, and four water molecules. In (C), the sodium ion bound to CCR5 interacts with D2.50 (bivalent coordination), N7.49, and two water molecules. In (D), the sodium ion bound to CCR5 interacts with the carbonyl group of G3.35, the hydroxyl group of S2.49 and three water molecules. D2.50 is in the second coordination shell and interacts with the sodium ion through a water molecule. In the four plots, sodium is shown as a yellow sphere. Water molecules within the sodium binding pocket are shown as spheres. Those within 3 Å of the sodium ion are red, otherwise they are pink. Side chains interacting with the sodium ion or contributing to the sodium binding site are shown as sticks. For clarity purpose, hydrogen atoms are not shown except the hydrogen of the hydroxyl group of S2.49 in (D) which is H-bonded to S3.38.

Typical snapshots of the sodium ion bound to CXCR4 and CCR5 are displayed in Fig 4. In CXCR4, the ion is coordinated to four protein atoms (D2.50:OD1, N3.35:OD1, S3.39:OG, N7.45:NE2) and to the oxygen atom of one water molecule. The position of the H7.45 ring is maintained by face-to-edge interaction with W6.48 in the *g*^*+*^ rotameric state. The second closest water molecule links the D2.50:OD2 atom to the backbone distortion of TM7 (H7.45:O and N7.49:N), while the third one links the D2.50:OD2 atom to the N1.50:OD1 and N7.49: ND2 atoms. Two different binding modes of the sodium ion to CCR5, obtained from classical MD simulations, are shown in Fig 4B and 4C. In Fig 4B, the sodium ion interacts with the OD1 atom of D2.50, the NE2 atom of H7.45 in the *g*^*-*^ conformation and four water molecules, an interaction resulting in a coordination number of six. In Fig 4C, H7.45 is now in the *trans* conformation. The sodium ion has a bivalent coordination with the OD1 and OD2 atoms of D2.50, it also interacts with the OD1 atom of N7.49 and with two water molecules, resulting in a coordination number of five.

**Fig 5:**
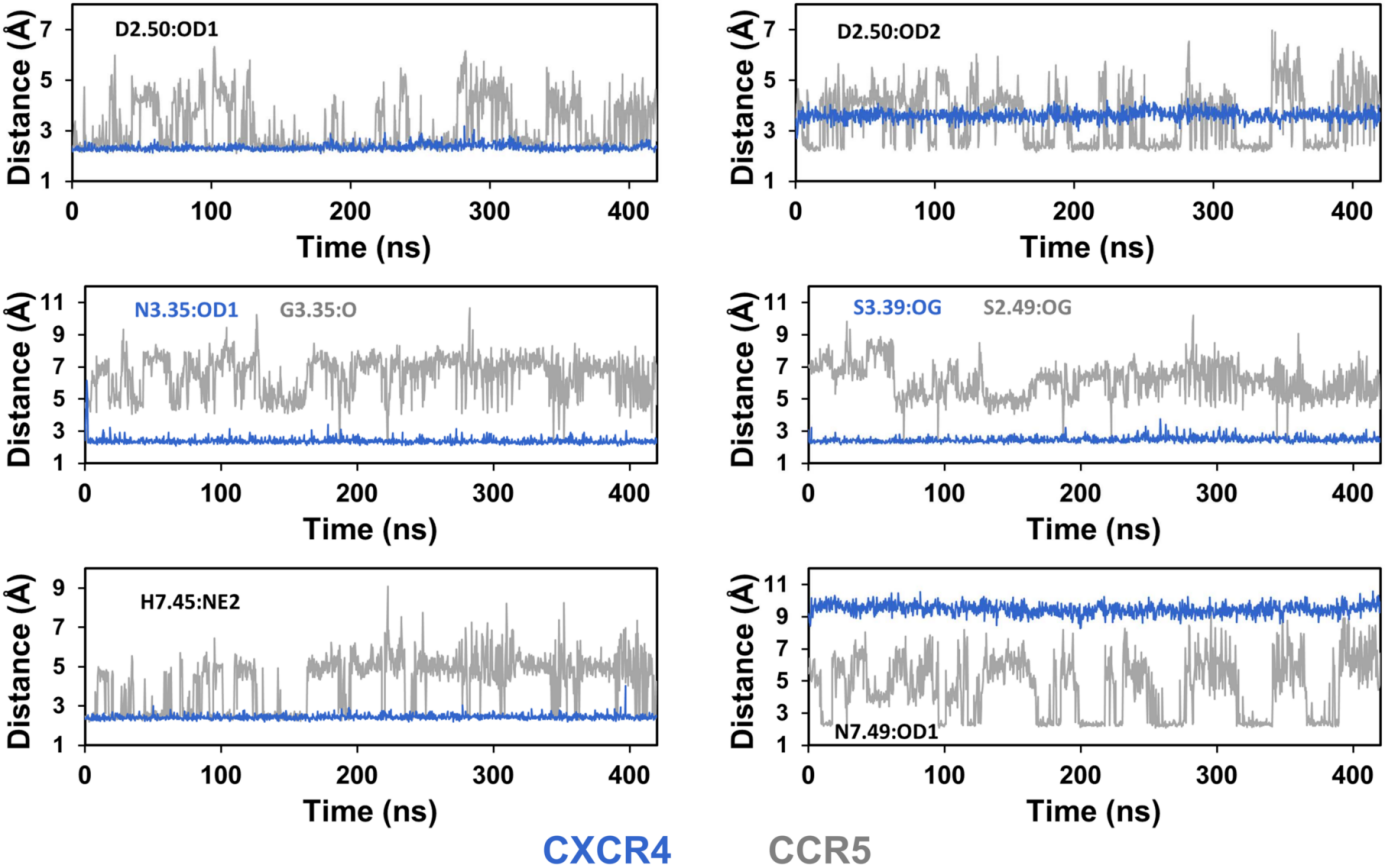
Distances of the sodium ion to putative coordinating atoms of CXCR4 and CCR5 during MD simulations. The putative coordinating atoms are D2.50:OD1 and D2.50:OD2 for both receptors (top panel), N3.35:OD1 and S3.39:OG for CXCR4, G3.35:O and S2.49:OG for CCR5 (middle panel), and H7.45:NE2 and N7.49:OD1 for both receptors (bottom panel). The graphs correspond to the trajectories shown in Fig 3C, with blue and grey curves for CXCR4 and CCR5, respectively.

To better characterize the environment of the sodium ion in CXCR4 and CCR5, we measured the distances between the ion and the putative protein coordinating atoms (Fig 5). Receptor coordinating atoms include oxygen atoms from carbonyl, carboxyl, and hydroxyl groups, and the nitrogen atom with lone-pair electrons from imidazole rings. This nitrogen corresponds to the NE2 atom since histidine residues have been modeled in the most frequent tautomeric form with the hydrogen atom on the ND1 atom. In CXCR4, the sodium ion remained within coordination distance of the D2.50:OD1, N3.35:OD1, S3.39:OG and N7.45:NE2 atoms for at least 98% of the trajectories (Table 1). No contact was observed with the D2.50:OD2 or the N7.49:OD1 atoms. In contrast to CXCR4, the coordinating atoms of the sodium ion in CCR5 included the OD1 and OD2 atoms of D2.50, the NE2 atom of N7.45 and the OD1 atom of N7.49. Contacts with these residues could last several tenths of nanoseconds but were not stable on the sub-microsecond timescale. The ion moved between several sub-sites and its coordination was reorganized within and between trajectories. The sodium ion could have monovalent or bivalent coordination with the OD1 and/or the OD2 atoms of D2.50. It could also be coordinated with the NE2 atom of H7.45 and with the OD1 atom of N7.49 (these two interactions were mutually exclusive). In addition to the contacts displayed in Fig 4B and 4C, other modes of interaction were observed, for example, with water molecules bridging the sodium ion and the D2.50 side chain, but these modes usually involved at least one contact with D2.50, H7.45 or N7.49. Finally, the N3.35G and A2.49S mutations created a cavity in which the G3.35:O and S2.49:OG atoms might act as an additional binding site when S2.49 was in the *trans* orientation. However, only transient contacts with these atoms were observed in the CCR5 trajectories (Fig 5). Analysis of the contact frequencies highlights the variability in the sodium binding mode of CCR5 (Table 1). For both CXCR4 and CCR5, during the contacts, the distances between the sodium ion and the coordinating atoms were similar to those observed in the crystal structures (Table 2).

**Table 1:** Frequency of the contacts between the sodium ion and the receptor coordinating atoms.

Atom	CXCR4	CCR5
D2.50:OD1	0.99 ± 0.01	0.59± 0.24
D2.50:OD2	0.01 ± 0.01	0.41 ± 0.12
N3.35:OD1/G3.35:O	0.98 ± 0.02	< 0.1
S3.39:OG/S2.49:OG	0.99 ± 0.01	<0.1
H7.45:NE2	0.99 ± 0.01	0.68 ± 0.20
N7.49:OD1	0	0.17 ± 0.13

The data represent the frequency of frames with the sodium ion within contact distance of the receptor coordinating atoms (threshold of 3 Å). Data reported are the average ± standard deviations of the CXCR4 and CCR5 replicates.

**Table 2:** Average distances of the sodium ion to receptor coordinating atoms.

Atom	CXCR4	CCR5	OPRD	PAR1	β1AR	A2AR
D2.50:OD1	2.3 ± 0.1	2.4 ± 0.2	2.2	3.6	2.6	2.5
D2.50:OD2	> 3	2.4 ± 0.2	3.8	2.8	4.1	3.6
N3.35:OD1	2.4 ± 0.2		2.4	*via* H_2_O		
S3.39:OG	2.4 ± 0.2		2.4	2.6	2.4	2.5
H7.45:NE2	2.4 ± 0.2	2.5 ± 0.2				
N7.45:OD1			*via* H_2_O		*via* H_2_O	*via* H_2_O
S7.45:OG				*via* H_2_O		
N7.49:OD1	> 3	2.3 ± 0.2	*via* H_2_O		*via* H_2_O	*via* H_2_O
D7.49:OD2				2.4		

Average distances ± standard deviations (Å) measured during the MD trajectories when the sodium ion was within contact distance of the receptor coordinating atoms (threshold of 3 Å). The distances of the sodium ion to coordinating atoms in OPRD (4N6H), PAR1 (3VW7), B1AR (4BVN), and A2AR (4EIY) are given for comparative purpose.

**Fig 6:**
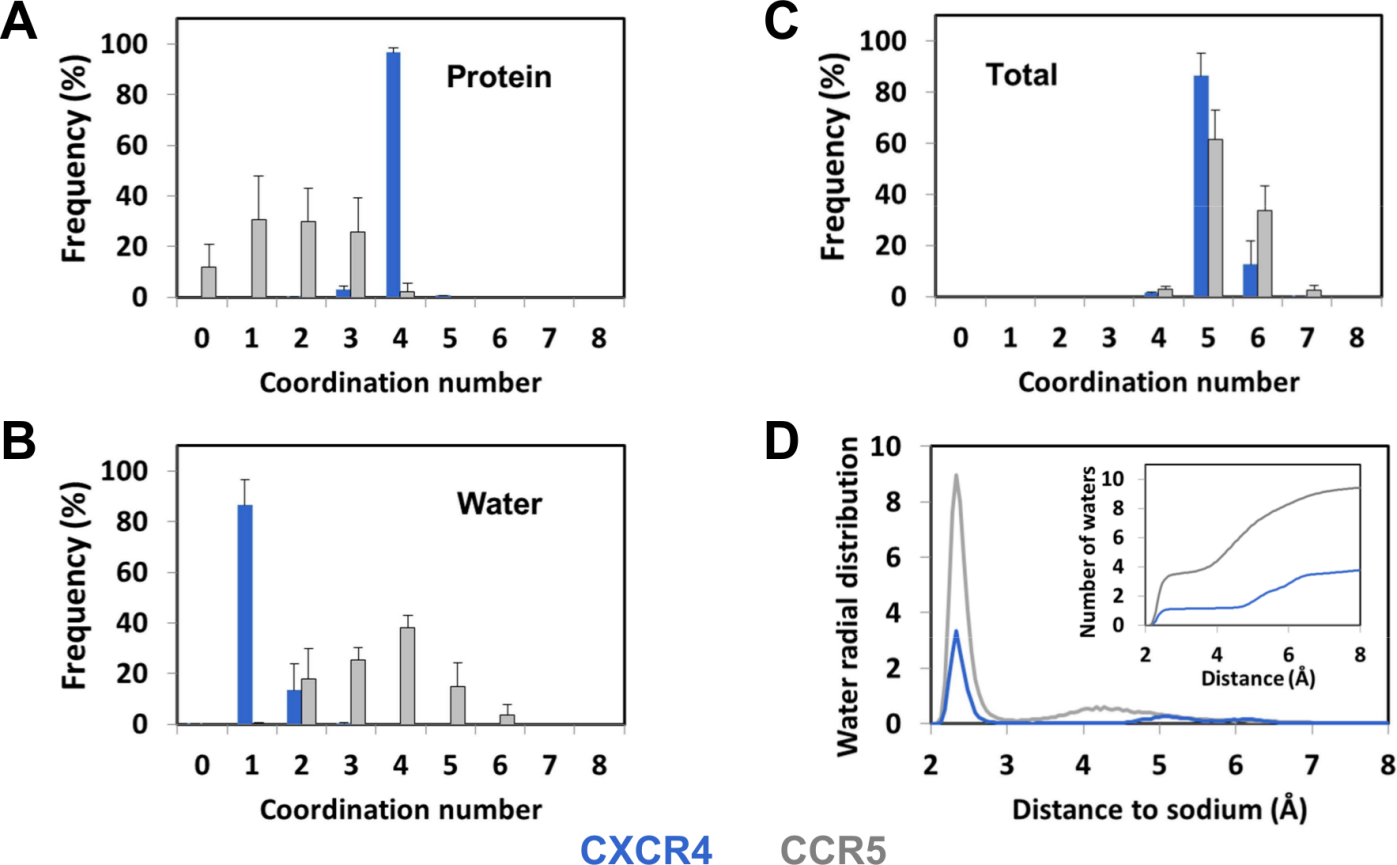
Coordination of the sodium ion bound to CXCR4 and CCR5 during MD simulations. (A) Distribution of the number of interactions between the sodium ion and coordinating atoms from the protein (carbonyl, carboxyl and hydroxyl groups, NE2 from His). (B) Distribution of the number of interactions between the sodium ion and water molecules. (C) Distribution of the total coordination number of the sodium ion. In (A-C), the threshold for interaction was 3Å. (D) Radial distribution of the water molecules around the sodium ion during the MD simulations. The insert represents the integrated radial distribution and gives the number of water molecules as a function of the distance to the sodium ion. Data represent the average of the replicates (± standard deviations in A-C). Data for CXCR4 and CCR5 are shown in blue and grey, respectively.

The analysis of the number of protein atoms coordinated to the sodium ion during the MD simulations (Fig 6) corroborates the diversity of the sodium binding modes in CCR5. This number usually varied between 1 and 3 with similar weights of about 30% but, in about 10% of the frames, no direct contact was observed. In contrast with CCR5, the sodium ion in CXCR4 was coordinated to four protein atoms in 97 ± 2% of the trajectory frames. We also investigated the number of water molecules in the vicinity of the sodium ion. In approximately 85% of the CXCR4 trajectory frames, the coordination of the sodium ion was completed by the oxygen atom from a single water molecule (Fig 4A). In the remaining frames, a second water molecule was present in the first coordination shell of the ion. This water molecule, which was hydrogen-bonded to N3.35:ND2 and L2.26:O upon interaction with the sodium ion, was located between TM2, TM3, and TM4. For CCR5, the number of water molecules in the first shell of the sodium ion varied from 2 to 5. The total coordination number (Fig 6C) did not display such variability with an average value of 5.4 ± 0.2 for CCR5, to be compared to 5.1 ± 0.1 for CXCR4. These values are consistent with data mining analysis of the sodium environment in proteins [26].

Finally, to further characterize the sodium binding site, we calculated the radial distribution function of water around the sodium ion in CXCR4 and CCR5 (Fig 6D). Comparison of these distribution functions highlights the differences between the CXCR4 and CCR5 sodium binding pockets. For CXCR4, in addition to the water molecules in the first coordination shell of the ion at a distance of about 2.5 Å, only two water molecules could be present at a distance of about 5 and 6.5 Å to the sodium ion, as observed in the snapshot displayed in Fig 4A. For CCR5, eight to nine water molecules were present in the first two shells. These differences can be explained by the difference in the sizes of the internal pocket in the vicinity of D2.50 in CXCR4 and CCR5. Indeed, the size increased from 90 Å^3^ in CXCR4 to 236 Å^3^ in CCR5, a pattern which is consistent with the changes in side chain volume upon the N3.35G (54 Å^3^) and S3.39G (29 Å^3^) mutations. The wider pocket in CCR5 can accommodate more water molecules than CXCR4 and does not constraint the sodium ion which can move by up to 3–4 Å during the simulations (Fig 3). These changes in the size of the sodium pocket result in static and dynamic sodium binding modes in CXCR4 and CCR5, respectively. It is worth noting that, in spite of the high mobility of the sodium ion in CCR5, an egress of the ion was not observed during the simulations.

### Alternative sodium binding site in CCR5

**Fig 7:**
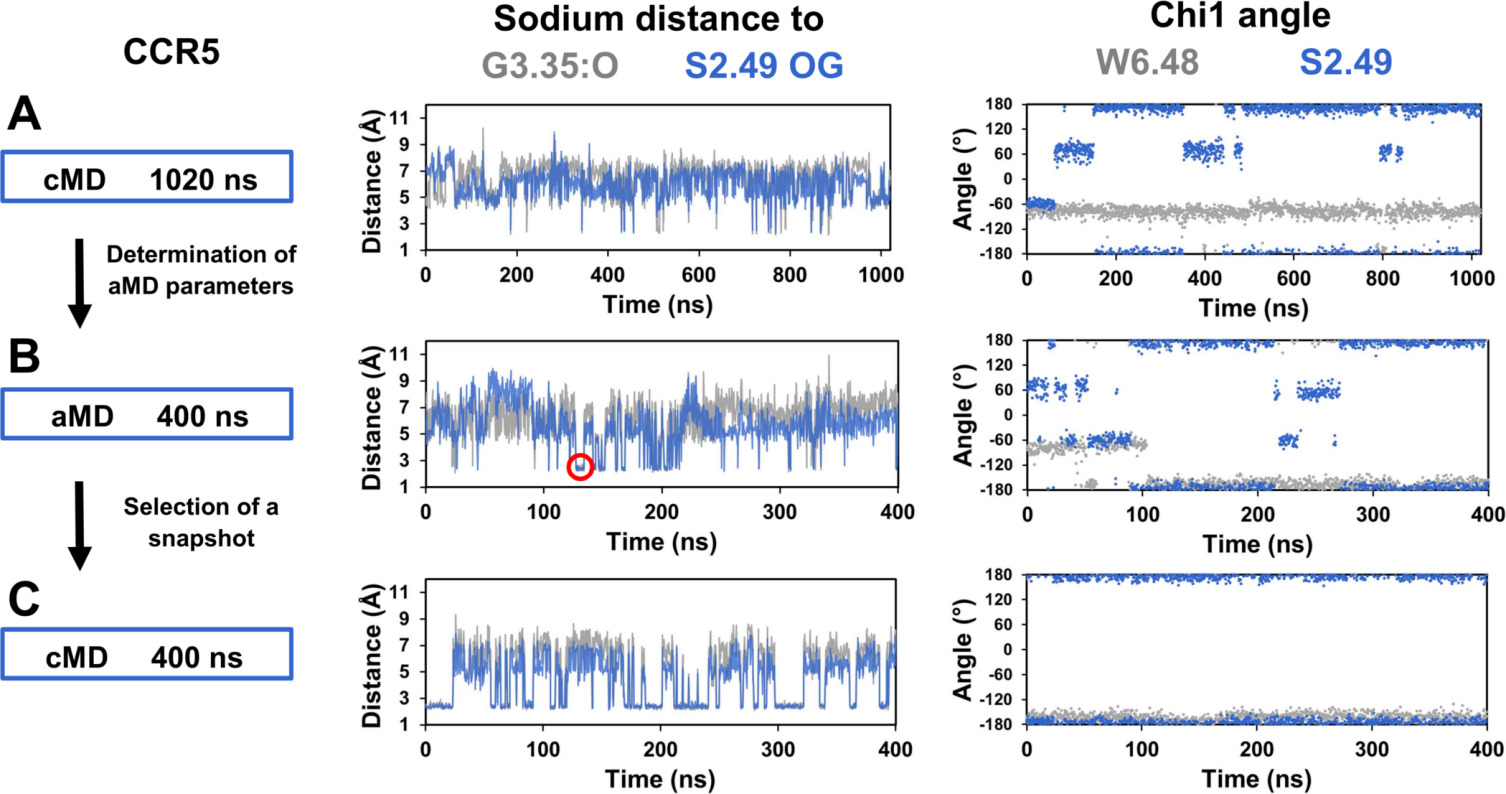
Alternative sodium binding site in CCR5. In (A), classical MD simulations (cMD) of CCR5 was extended to 1.0 microsecond. Energy parameters from this trajectory were used to calculate acceleration parameters for the accelerated MD simulations (aMD) shown in (B). A snapshot with the sodium ion coordinated with the G3.35:O and S2.49:OG atoms (indicated by a red circle in the distance panel) was selected and used to initiate the classical MD simulations shown in (C). The left panel summarizes the protocol. The central panel shows the distances of the sodium ion to the G3.35:O and S2.49:OG atoms, colored in grey and blue, respectively. The right panel shows the chi1 dihedral angles of W6.48 and S2.49, colored in grey and blue, respectively.

The split between homeostatic and inflammatory chemokine receptors is characterized by the A2.49S mutation, which lines the sodium binding pocket. In CCR5, when S2.49 is in the *trans* rotameric state, it faces the sodium binding site at a distance of 2.8 Å from the carbonyl group of G3.35. This geometry could provide an additional binding site to the sodium ion. However, we observed only transient escapes of the sodium ion toward this putative site (Fig 5). We extended this simulation to 1.0 microsecond but failed to observe stable binding of the ion to the putative site, albeit the *trans* orientation of S2.49 was stable (Fig 7). In order to obtain a larger sampling of the receptor conformational spaces, we carried out accelerated molecular dynamics (aMD) simulations [27] of CCR5. In these accelerated trajectories, the RMSD of the sodium ion, 3.0 ± 0.9 Å, indicated high fluctuations of the sodium ion within the binding pocket on the nanosecond time scale. Frequent interactions of the ion with both G3.35:O and S2.49:OG were observed and could last several nanoseconds, after rotamerization of W6.48 to the *trans* conformation (Fig 7B). In order to determine whether the alternative binding site reached during aMD simulations could remain stable during classical MD simulations, a snapshot of the aMD trajectory with the sodium ion interacting with the G3.35:O and S2.49:OG atoms was selected. The system was energy minimized and used as starting coordinates for subsequent classical MD simulations (Fig 7C). In four out of five replicates, during several tens of nanoseconds (from 18 to 58 ns), the sodium ion remained at this position, and then could experience exchanges between the alternative and canonical sites that are distant of 3–4 Å (Fig 7C). In the alternative site (Fig 4D), the sodium ion is coordinated to the carbonyl oxygen of G3.35, the hydroxyl oxygen of S2.49 and three water molecules. In addition, S2.49 is H-bonded to S3.38, providing further stability to this configuration. D2.50 is now located in the second coordination shell and interacts with the sodium ion through one or occasionally two water molecules. We can note that W6.48, in the *trans* conformation, forms a trap that closes the sodium binding pocket. In this conformation, it cannot form the face-to-edge interactions with H7.45 that favor the interaction of the latter residue with the sodium ion. The rotameric state of W6.48 might explain the differences observed between the simulations restarted from the aMD snapshot and the initial MD simulations in which W6.48 remained in the crystal structure conformation (Fig 7).

## Discussion

In this article, we seek to identify the key residues that drove the evolution of chemokine receptors. Nested PCA analysis of sequence covariation matrices (Fig 1) highlighted three positions (i.e., 3.35, 7.45, and 2.49) that are part of the allosteric sodium binding pocket [18]. Mutation of at least one of these positions was crucial at each hierarchical step that led from class A receptors to the split between homeostatic and inflammatory chemokine receptors. This observation prompted us to investigate the history of these positions and their structural and functional roles in prototypical chemokine receptors.

Sodium has been shown to be an important regulator of a wide variety of class A GPCRs, acting as a negative allosteric modulator [18]. This allosteric role has been confirmed by the presence of a sodium ion bound in a conserved position in several high resolution structures of GPCRs [21, 22, 28, 29]. The overall binding cavity is conserved within class A GPCRs and involves highly conserved residues, especially D2.50 (fully conserved), but also S/T3.39 (80% conserved), and N7.45 (67% conserved), a pattern suggesting that sodium binding may be a general property of class A GPCRs [18]. A recent MD investigation of the free energy profiles and kinetics of sodium binding to 18 GPCRs revealed a conserved sodium binding mechanism [19]. In addition to its documented role as a negative allosteric modulator, the sodium ion might contribute to the mechanisms of receptor activation [30, 31], to voltage sensing [32], and to biased signaling [21, 33]. Moreover, presence of a sodium binding site might have contributed to the evolutionary success of class A GPCRs [18]. In view with this latter role, it is noteworthy that expansion of P2.58 receptors in vertebrates with the emergence of the CHEM and PUR sub-families is subsequent to the appearance of the N3.35 pattern in the SO receptors of chordates (Fig 2). In the sodium bound crystal structures of two P2.58 receptors, OPRD and PAR1, N3.35 is involved in the first or in the second coordination shell of the sodium ion [21, 22].

The divergence that led to the chemokine receptors is characterized by a specific mutation at position 7.45 (Fig 1). This position is preferentially an Asn residue in class A receptors (67%) but a His, or in a few cases, an Arg residue, in chemokine receptors. Oldest chemokine receptors, CXCR4 and ACKR3/CXCR7, present in the lamprey [3], possess a histidine at this position, indicating that the divergence to chemokine receptors involved the N7.45H mutation. Our molecular dynamics simulations of CXCR4 and CCR5 indicate that H7.45 participates in sodium binding. Indeed, in the neutral state, one nitrogen atom of the imidazole ring (usually NE2) has a lone-pair of electrons that allow His to act as a Lewis Base to form coordination complexes. Coordination to divalent ions (e.g., Fe^++^, Zn^++^, Ni^++^, Cu^++^) is frequently found in proteins [26]. Coordination to the sodium ion is less frequent but is also observed either in model systems [34] or in proteins [26]. The distance of about 2.5 Å (Table 2) that we observed is in agreement with statistical analysis of the Cambridge Structural Database [35]. In chemokine receptors (Fig 2B), H7.45 can only be substituted by Arg, which allows direct salt bridge interaction with D2.50 to stabilize inactive structure.

We and others previously noted that the split between homeostatic and inflammatory chemokine receptors is characterized by an Ala to Ser mutation at position 2.49 [17, 36]. This position strongly covaries with position 3.35 (second component in Fig 1C). Position 3.35, which is a conserved Asn in 70% of P2.58 receptors, is Gly in the small subset of inflammatory chemokine receptors. This latter mutation is striking because of the importance of N3.35 in sodium binding and its role in the stability of the receptor inactive state. In the homeostatic receptors CXCR4 [23] and CXCR3 [24], the mutation of N3.35 to Ser or Ala induces constitutive G protein and β-arrestin activity. Among inflammatory chemokine receptors, the direct effect of sodium ions on receptor activity has been experimentally verified on CCR3 [37], which possesses a Gly residue at position 3.35, a configuration indicating that these receptors maintain the ability to bind sodium ions. With this regard, the presence of a His residue at position 7.45, involved in sodium binding, might explain the maintained sodium binding. Nevertheless, MD simulations highlight dramatic differences in the sodium binding sites of CXCR4 and CCR5.

In CXCR4, the tightly bound sodium ion is coordinated to four protein atoms from D2.50, N3.35, S3.39 and H7.45 and to one or occasionally two water molecules. The contacts with these protein atoms are maintained during at least 98% of the trajectory (Table 1). Such pentameric coordination with four receptor atoms in the first coordination shell has not been observed yet in GPCR crystal structures, but examples in other protein families have been reported [38]. The stability of the sodium binding site is reinforced by the face-to-edge interactions between H7.45 and W6.48 (Fig 4A). This geometry should strongly stabilize the inactive state of CXCR4 in the absence of agonists. We verified that the four residues involved in sodium coordination are conserved from *P*. *Marinus* (Sea Lamprey, Uniprot access number: Q802H1) to humans.

A very different pattern is observed in CCR5. The absence of side chains at positions 3.35 and 3.39 creates a large water-filled pocket (Fig 6D). Sodium is highly mobile within this pocket and experiences different binding modes that depend on the rotameric states of W6.48 and H7.45 and involve two to five water molecules (Fig 6). Different binding modes of the sodium ion within the allosteric binding pocket have also been reported in MD simulations of A2A adenosine receptor [39], opioid receptors [30, 40] and dopaminergic D2 receptor [41]. In these simulations, the sodium ion remains in the close vicinity of residues 2.50, 3.39, or 7.45. Similarly, in the CCR5 simulations started from a model based on the crystal structure, the sodium ion remains in the vicinity of residues 2.50, 7.45 and 7.49 (Fig 5). The alternative sodium binding site, located 3–4 Å apart, is observed only under a specific MD protocol (Fig 7). Albeit the possibility of an aMD skew cannot be ruled out, we note that the alternative site involves G3.35 and S2.49, the two residues crucial for the emergence of the inflammatory chemokine receptors (Fig 1). Interestingly, its relative stability might be related to the rotameric orientation of W6.48 (Fig 7). W6.48 is a crucial microswitch for receptor activation following agonist binding [42]. The alternative site might thus be stabilized by ligand binding or environmental conditions. Moreover, in contrast with the sites that involve residues from TM7 and collapse upon receptor activation, this site might be maintained upon activation.

The differences in the sodium binding modes (Figs 3–6) prompt the question of their contribution to the physiological roles and mechanisms of action of CXCR4 and CCR5. In addition to different physiological functions in homeostasis and inflammation, these receptors differ by several aspects of molecular and cellular regulation: (1) Ligand selectivity: CXCR4 is monogamous with a single native chemokine ligand, CXCL12. CCR5 is highly promiscuous with ten chemokine ligands [1]; (2) Pre-coupling with G proteins: Pre-coupling of CCR5 with nucleotide free G proteins leads to two receptor populations. Pre-coupled receptors bind chemokine ligands with Kd values in the nanomolar range, close to physiological concentrations, while free receptors have a much lower affinity for chemokines, which prevent chemokine inhibition of HIV-1 entry [43]. A unique Kd in the nanomolar range is observed for CXCL12 binding to CXCR4 [44]; (3) Constitutive activity: CCR5 is partially constitutively active for both the Gαi pathway and the β-arrestin mediated internalization [45, 46]. Wild type CXCR4 undergoes constitutive internalization by a β-arrestin pathway [47, 48] but constitutive G protein activity of CXCR4 has been observed only upon mutation of N3.35 to Ser or Ala [23]. In spite of these differences, it is interesting to note that, as observed for other chemokine receptors, CXCR4 and CCR5 do not possess an ionic lock [42] between position 3.50 (Arg in both receptors) and position 6.30 (Lys and Arg in CXCR4 and CCR5, respectively).

CXCR4 is not only involved in homing of hematopoietic stem cells but is also a major player of embryonic development, especially neuronal development [2, 49, 50]. Evolutionary analysis indicates that CXCR4 is the ancestor of chemokine receptors and suggests that the developmental role of CXCR4 is the initial function that has emerged in evolution [4]. Constitutive internalization, mediated by β-arrestins and related to receptor recycling, is necessary for high responsiveness of migrating cells upon stimulation [51]. A stringent control of the activation of Gαi (related to migration) by CXCR4 to avoid erroneous activation in the absence of CXCL12 stimulation is mandatory for the role of CXCR4 in neuronal development. Release of the ionic lock joined to tight sodium binding might have been a solution for this challenge. Interestingly, the urotensin II receptor (UR2R) which appeared in chordates (Fig 2) has properties reminiscent of CXCR4, including chemotaxic properties and involvement in cancer metastasis [52], along with location in the central nervous system [53]. It possesses a polar Thr residue at position 3.35, and no ionic lock from *B*. *floridae* to humans, suggesting that tightening of sodium binding and absence of ionic lock might have been mandatory for emergence of chemotaxic properties in P2.58 receptors.

In CCR5, the loosening of the sodium binding site results in different binding modes of the sodium ion within the sodium pocket. Such variability may be important for the functionality of CCR5 which induces biased responses upon binding to different chemokine ligands [13]. Indeed, in several receptors, altering sodium binding induced biased signaling [21, 33, 54]. Interestingly, in the δ-opioid receptor, the N3.35V mutation facilitates the mobility of the sodium ion within the allosteric binding pocket [32] and augments constitutive β-arrestin-mediated signaling [21]. In addition, the capability to maintain sodium binding at the alternative site upon activation or pre-activation might contribute to the pre-coupling of CCR5 with G proteins. Further studies will be required to investigate the putative function of this site which might be involved in biased signaling or in G protein pre-coupling.

Table 3 reports the positions involved in sodium binding within the chemokine receptor family. The wide variety of sodium coordination in a small receptor sub-family with high sequence identity (> 30%) contrasts with the usual conservation of sodium binding mechanism within a receptor sub-family and the high conservation of the S/T3.39 and N7.45 patterns in class A GPCR (see above). This high variability in the sodium binding mechanism of chemokine receptors strongly supports the assumption that the sodium ion has a major role in the mechanism of action and regulation of these receptors.

**Table 3:** Evolution of the sodium binding site in the chemokine receptor family.

Receptors	Positions ^a^					*N* ^b^	Genomes ^c^			
	2.49	3.35	3.39	6.48	7.45		L	S	Z	H
ACKR3/CXCR7	A	N	S	W	H	2	••	•	•	•
CXCR4	A	N	S	W	H	1	•	•	•	•
CXCR5	A	N	S	W	H	1		•	•	•
CXCR3	A	N	G	W	H	10			•	•
CCR10	A	S	G	Q	R	2			•	•
CXCR2	A	N	G	W	H	7			•	•
CXCR1	A	N	G	W	H	3		••	•	•
CCR6	A	N	G	Q	H	1		•	••	•
CXCR6	A	N	S	Q	R	1		•		•
ACKR4/CCRL1	A	N	G	Q	H	3			•	•
CCR9	A	N	C	Q	H	1		•	•	•
CCR7	A	S	G	Q	R	2		•	•	•
XCR1	S	S	S	W	H	1		•	•	•
ACKR2/CCBP2/D6	S	N	G	W	H	11				•
CX3CR1	S	G	S	W	H	1			•	•
CCR8	S	G	S	W	H	1		•	•	•
CCR4	S	G	G	W	H	2				•
CCR2	S	G	G	W	H	8				•
CCR5	S	G	G	W	H	10				•
CCRL2	S	G	E	W	H	2				•
CCR1	S	G	E	W	H	11				•
CCR3	S	G	E	W	H	14				•

^a^ The residues at the indicated positions were obtained from the human sequences.

^b^ *N* represents the number of binding chemokines for the human receptors [1].

^c^ The presence of orthologs of human chemokine receptors in different genomes is based on Nomiyama’s data [3]. L: Sea lamprey (jawless fish), S: Elephant shark (cartilaginous fish), Z: zebra fish (bony fish); H: Human. Double symbols indicate two orthologs. The receptors that are specific to fish species and not present in humans are not indicated.

We note that that only three homeostatic chemokine receptors can provide 4 protein atoms for the sodium coordination: (1) CXCR4, (2) ACKR3/CXCR7 which is an atypical chemokine receptor that works in tandem with CXCR4 and acts as a scavenger to maintain CXCL12 gradient [55], and (3) CXCR5, a receptor important for secondary lymphoid tissue orchestration and lymphoid neogenesis [56]. These receptors, which bind one or two chemokines (Table 3), are among the most ancient chemokine receptors since they appeared in jawless or cartilaginous fishes [4]. In most other homeostatic receptors, position 3.39 is a glycine. In CXCR6, S3.39 is present but position 7.45 has been mutated to Arg. In CCR9 with a conservative S3.39C mutation, the highly conserved W6.48 is mutated to Gln, which prevents tight packing of H7.45. Thus, tight sodium binding seems related to initial functions of most ancestral chemokine receptors (e.g., neuronal development) and this constraint was released with the emergence of chemokine receptors with different physiological functions.

The loose sodium binding pocket observed in CCR5 may also be an extreme case. This property is related to the G3.35/G3.39 pattern which is shared only by CCR2 and CCR4, closely related to CCR5 (Fig 2). Other inflammatory receptors possess the S3.39 pattern (receptors present in fish), the E3.39 pattern (CCR1, CCR3, and CCRL2) or the N3.35 pattern (ACKR2/CCBP2), which provides additional protein oxygen for sodium coordination (Table 3).

Another important observation in Table 3 is the absence of the S3.39 pattern in promiscuous receptors. Receptors with the S3.39 pattern bind only one or two chemokine ligands. Promiscuous receptors have either a glycine or a glutamic acid at this position, indicating different sodium binding modes. In the chemokine/receptor system, promiscuity is linked to signaling bias. Chemokines do not induce similar response upon binding to a promiscuous receptor, but constitute an example of naturally biased ligands [13]. In several receptors, biased signaling was induced by altering residues involved in sodium binding. This has been observed, for example, in OPRD [21] and AT1 [54]. This suggests a relation between sodium binding and receptor promiscuity/biased signaling that will require experimental investigation.

In conclusion, the evolutionary approach that we have developed has pointed out a key role of mutations at the sodium binding site in the evolutionary pathway that led to the emergence and diversification of chemokine receptors. Molecular dynamics simulations have highlighted the dramatic changes in the mechanism of sodium binding between homeostatic and inflammatory chemokine receptors, exemplified by CXCR4 and CCR5, respectively. The tight binding site observed for CXCR4 is replaced by a loose binding pocket for CCR5, in which several binding modes are possible. These findings might be related to the specific features of CCR5: pre-coupling, constitutive G protein activity, promiscuous binding and biased signaling upon chemokine binding. The sodium binding site links the orthosteric ligand binding site and the G protein/β-arrestin binding site. Careful analysis of sodium binding properties of chemokine receptors will help the understanding of the mechanisms underlying biased signaling and the subsequent design of biased drugs.

## Methods

### Sequence analysis

The analyzed sequences included non-redundant sets of class A GPCRs from different genomes: *H*. *sapiens*, *D*. *rerio*, *B*. *floridae*, *C*. *elegans*, *N*. *vectensis*, and *T*. *adhaerens*. All the sets were prepared, aligned, and assigned as described previously [16]. In this analysis, *B*. *Floridae* was chosen as the representative chordate because it corresponds to a reference proteome (www.uniprot.org). The DARC/Duffy/ACKR1 atypical chemokine receptor was not included in the human set because of its low sequence identity with other chemokine receptors (< 16%) and loss of typical class A sequences patterns in TM1, TM2, TM3, and TM5, making it a remote outlier.

Neighbor joining trees were obtained with the MEGA5 software [57], using the Dayhoff matrix option and 500 bootstrap replicates. Sequence logos were obtained from the WebLogo site (web.logo.berkeley.edu) [58]. Covariation analysis was carried out on two sequence sets from humans: the non-redundant set of class A GPCRs (282 sequences) and the CHEM sub-family subset (46 sequences) [15]. The sequence analyses were performed using positions with less than 2% gap in the MSA (extended transmembrane helices). The MSAs that were used for sequence analysis are available as Supporting Information (S1, S2 and S3 Files for, respectively, the human class A set, the human CHEM set, and the SO receptors from the six species investigated). The GPCR positions were numbered by reference to the most conserved position *i*.50 in each helix *i*, according to Ballesteros’ numbering [59]. In CXCR4, the anchor residues are: N56, D84, R134, W131, P211, P254, and P299. In CCR5, the anchor residues are: N48, D76, R126, W153, P206, P250, and P 294.

### Covariation analysis

The covariation score between any two positions *i* and *j* in the alignment of length *N* was measured by the OMES method [60] which is suited to find sequence covariation related to family divergence [17]. The covariation matrix, denoted **COV,** was subsequently double-centered to give the matrix **S** according to the formula:

$$S=\left[I-\frac{1}{N}1\right]\times COV\times {\left[I-\frac{1}{N}1\right]}^{T}$$ (1)

where **I** denotes the *N* by *N* identity matrix, and **1** an *N* by *N* matrix of ones. The matrix **S** was subsequently analyzed by principal component analysis (specifically, by an eigen-decomposition) [20]:

$$S=U\Lambda U^{T}$$ (2)

where **U** and **Λ** are, respectively, the matrix of the eigenvectors and the diagonal matrix of the eigenvalues of **S**. The coordinate matrix (a.k.a., factor scores matrix [20,72]), denoted **F**, gives the coordinates of each position of the alignment on the principal component axes and is computed as:

$$F=U\Lambda^{\frac{1}{2}}.$$ (3)

The analysis was performed with functions written in the R programing language. They are available in the R package **Bios2cor** which can be found in the Comprehensive R Archive Network (cran.r-project.org).

### Molecular modeling

The models of CXCR4 (residues 28 to 319) and CCR5 (residues 19 to 313) with a bound sodium ion were built with MODELLER version 9.8 [61], using as templates the crystal structure of CXCR4 (PDB entry 3ODU) [11] and CCR5 (PDB entry 4MBS) [10], respectively, and the high resolution (1.8 Å) crystal structure of the δ-opioid receptor (PDB entry 4N6H) [21] that includes a bound sodium ion interacting with D2.50 and N3.35. The water molecules contributing to the coordination of the sodium ion were included in the models. The three templates correspond to receptors in inactive states. The stabilizing mutations present in the crystallized CXCR4 and CCR5 receptors were reversed to the amino acids present in the human wild type receptor. The ICL3 loops that were replaced by the lysozyme and rubredoxin in the crystallized CXCR4 and CCR5, respectively, were reversed to the human sequences of the corresponding receptors. The carboxyl group of D2.50 was deprotonated [62]. H7.45 which was completely buried from solvent was neutral. Its pKa was measured using Propka on the PDB2PQR web interface [63], in the presence and absence of sodium, and was below 4 in any case for both receptors. The hydrogen of the pyrimidole ring was located on ND1 which corresponds to the most frequent tautomer (HSD in the CHARMM topology file). Subsequently, the models were prepared for molecular dynamics simulations (MD) using the Charmm-gui interface [64]. The models were embedded in a palmitoyl-oleoyl-phosphatidyl-choline (POPC) lipid bilayer and solvated using the TIP3P model for water molecules [65], with all atoms represented explicitly. The charges were neutralized by adding chloride ions.

### Molecular dynamics simulations

Molecular dynamics simulations of the CXCR4 and CCR5 models embedded in an hydrated POPC bilayer were carried out using NAMD v2.9 MD software [66] and the CHARMM36 parameter set [67, 68]. They were performed using the HPC resources of IDRIS, granted by GENCI (www.genci.fr). The entire assembly was subjected to energy minimization for 5000 steps to remove close contacts between atoms. Equilibration of the system was carried out with a modified version of a protocol developed elsewhere [69]. The protocol included six interlinked equilibration steps in which harmonic restraints were gradually taken off to achieve a smooth relaxation, for a total of 1 ns. Then a 20 ns equilibration step was carried out under the same conditions as the production run to achieve stable conditions. In the first two equilibration steps, the NVT ensemble at 310 K and time step of 1 fs were used. The following equilibration and production steps were carried out at constant temperature (310 K) and pressure (1 atmosphere), using a 2 fs time-step for integration. The Particle Mesh Ewald method (PME) was used to calculate the electrostatic contribution to non-bonded interactions with a cutoff of 12 Å. The cutoff distance of the van der Waals interaction was 12.0 Å. The SHAKE algorithm was applied to the system. Each trajectory lasted 420 ns (20 ns for equilibration and 400 ns for production). Three and five replicates were carried out for CXCR4 and CCR5, respectively. The initial assembled models for the different runs and the scripts used for the equilibration and production steps are available as Supporting Information (S4 File)

A CCR5 trajectory displaying transient contacts to a putative alternative site was extended to 1 microsecond (Fig 7A). To obtain a more extensive sampling of the CCR5 conformational space, we carried out accelerated molecular dynamics simulations (aMD) [27]. This technique, based on an extended biased potential MD approach, has been shown to be an efficient way to enhance conformational sampling. It works by adding a dihedral potential boost to all dihedral angles in the system (dihedral boost) and, optionally, a total potential boost to all atoms in the system (dual boost) when the energies are below a threshold. The threshold energies were set to the average energies, *E*_*dihed_avg*_ and *E*_*pot_avg*_, computed from classical MD. The acceleration factors were calculated according to:

$$\alpha_{dihed}=\lambda \times \frac{E_{dihed\_avg}}{5}$$ (4)

$$\alpha_{pot}=\lambda \times N$$ (5)

where the acceleration parameter λ was chosen to be equal to 0.3 and *N* represents the number of atoms in the system [70, 71]. We used the 20–120 ns range of classical MD simulations to calculate the acceleration parameters. The snapshot obtained after 120 ns of classical MD simulations was used to initiate the accelerated simulation (S5 File). This technique allowed obtaining snapshots with the sodium ion interacting with the carbonyl oxygen of G3.35 and the hydroxyl oxygen of S2.49. A typical snapshot with the sodium ion in this position (S6 File) was used to restart the classical MD protocol to study the stability of this putative sodium site. Five replicates of 200 ns were performed and a representative run was extended to 400 ns.

Trajectories were analyzed with the VMD software [72] and the Bio3D R package [73], using in house-developed scripts. PYMOL (DeLano Scientific LLC, San Francisco, USA) was used for figure preparation. The frames obtained during the first 20 ns of the trajectories were not taken into account for the analysis of the coordination and hydration of the sodium ion.

### Software availability

The R package **Bios2cor,** developed for covariation analysis, can be downloaded from the Comprehensive R Archive Network (http://cran.r-project.org).

## Supporting information

S1 File Multiple sequence alignment of the non-redundant set of human class A GPCRs used for sequence analysis. Sequences are named by their Uniprot identifier.
(AA) Click here for additional data file.

S2 File Multiple sequence alignment of the human CHEM receptors used for sequence analysis. Sequences are named by their Uniprot identifier.
(AA) Click here for additional data file.

S3 File Multiple sequence alignment of the SO receptors used for sequence analysis. Receptors are from *H*. *sapiens*, *D*. *rerio*, *B*. *floridae*, *C*. *elegans*, *N*. *vectensis*, and *T*. *adhaerens*. Sequences are named by their Uniprot identifier.
(AA) Click here for additional data file.

S4 File Classical MD simulations. Initial CXCR4 and CCR5 models used for the different runs of classical MD simulations and NAMD scripts used for the equilibration and production steps.
(7Z) Click here for additional data file.

S5 File Accelerated MD simulations. Configuration of CCR5 that initiated the accelerated MD simulations and NAMD script used to run them.
(7Z) Click here for additional data file.

S6 File Restarted MD simulations. Snapshot of CCR5 obtained during the accelerated MD simulations and used to restart classical MD simulations.
(7Z) Click here for additional data file.
